# A biomimetic fly photoreceptor model elucidates how stochastic adaptive quantal sampling provides a large dynamic range

**DOI:** 10.1113/JP273614

**Published:** 2017-05-17

**Authors:** Zhuoyi Song, Mikko Juusola

**Affiliations:** ^1^ Department of Biomedical Science University of Sheffield Sheffield S10 2TN UK; ^2^ State Key Laboratory of Cognitive Neuroscience and Learning Beijing Normal University Beijing 100875 China

**Keywords:** fly photoreceptor, gain control, large dynamic range, light adaptation, phototransduction, quantum sampling, stochastic adaptive sampling

## Abstract

Light intensities (photons s^–1^ μm^–2^) in a natural scene vary over several orders of magnitude from shady woods to direct sunlight. A major challenge facing the visual system is how to map such a large dynamic input range into its limited output range, so that a signal is neither buried in noise in darkness nor saturated in brightness. A fly photoreceptor has achieved such a large dynamic range; it can encode intensity changes from single to billions of photons, outperforming man‐made light sensors. This performance requires powerful light adaptation, the neural implementation of which has only become clear recently. A computational fly photoreceptor model, which mimics the real phototransduction processes, has elucidated how light adaptation happens dynamically through stochastic adaptive quantal information sampling. A *Drosophila* R1–R6 photoreceptor's light sensor, the rhabdomere, has 30,000 microvilli, each of which stochastically samples incoming photons. Each microvillus employs a full G‐protein‐coupled receptor signalling pathway to adaptively transduce photons into quantum bumps (QBs, or samples). QBs then sum the macroscopic photoreceptor responses, governed by four quantal sampling factors (limitations): (i) the number of photon sampling units in the cell structure (microvilli), (ii) sample size (QB waveform), (iii) latency distribution (time delay between photon arrival and emergence of a QB), and (iv) refractory period distribution (time for a microvillus to recover after a QB). Here, we review how these factors jointly orchestrate light adaptation over a large dynamic range.

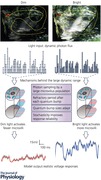

**Abbreviations**
GPCR: G‐protein‐coupled receptor
LIC: light‐induced current
QB: quantum bump
R1–R6: six outer photoreceptors
RandPam: random photon absorption model
TRP: transient receptor potential
TRPL: transient receptor potential like
PLC: phospholipase C

## Introduction

Vision starts from phototransduction; a photoreceptor absorbs photons (input) from the environment and transduces them into its electrical signals (output). For diurnal animals, a critical challenge facing phototransduction is the huge difference between the input and output ranges. Light intensities (photons s^–1^ μm^–2^) in a natural scene can span several orders of magnitude from shaded foliage to direct sunlight, in contrast to a photoreceptor's 30–60 mV electrical output range (Warrant & McIntyre, 1992). How to encode reliably and rapidly the large intensity variations with a limited output range is a real engineering challenge (Reinhard & Devlin, 2005). Photoreceptors of diurnal animals surpass man‐made light sensors in achieving a large dynamic range, as their sensitivity – or input–output gain – constantly adapts so that their signals are neither buried into background noise nor saturated by daylight (light adaptation/gain control) (van Hateren, 1997).

Resolving the inner workings of light adaptation has long fascinated both experimentalists and theoreticians. Early experimental work on the eye focused upon how various physical mechanisms regulate day/night vision sensitivity. These included, for example, changes in the pupil size or photopigment contents (Stavenga & Kuiper, 1977), and transitions from rod to cone pathways (Schultze, 1866). More recently, experimental eye research has shifted to the molecular dynamics, exploring rhodopsin bleaching (Minke, 1987), protein translocation (Hardie, 2003; Cronin *et al*. 2004) and Ca^2+^ feedbacks (Yau, 1991) as means to regulate phototransduction gain. Meanwhile, theoreticians have proposed optimization strategies for light adaptation from multiple perspectives, including redundancy reduction (Attneave, 1954; Barlow, 1961), information maximization (Atick, 1992; van Hateren, 1992) and predictive coding (Kretzmer, 1952; Srinivasan *et al*. 1982). However, the field has lacked a unified framework, which would link abstract theoretical principles to detailed neuronal mechanisms (Rieke & Rudd, 2009). So there has been a real need for transformative computational models, which would respond to photon inputs as real photoreceptors do, revealing the neural implementations of the theoretical principles.

A *Drosophila* R1–R6 photoreceptor is an attractive neural system for computational modelling to study light adaptation mechanisms, for two good reasons. Firstly, a fly photoreceptor achieves a very large dynamic range (French *et al*. 1993). Unlike vertebrate eyes, which use rods and cones to see dim and bright light separately (Schultze, 1866), a single R1–R6 photoreceptor can respond to the whole range of light intensities, from single to billions of photons. Secondly, R1–R6 photoreceptors are well studied, with a breadth of accumulated knowledge about their phototransduction cascades and an abundance of high‐quality electrophysiological data for model testing (Juusola & Hardie, 2001a; Hardie & Postma, 2008; Hardie & Juusola, 2015).

By using a novel bottom‐up biomimetic approach, we recently constructed a virtual *Drosophila* R1–R6 photoreceptor, which implements its real counterpart's structural and functional sampling constraints (Song *et al*. 2009, 2012). The model showed how stochastic adaptive photon sampling enables fly photoreceptors to achieve a large dynamic encoding range (Song *et al*. 2012; Song & Juusola, 2014). Here, we review how four stochastic quantal sampling factors (limitations), together with a fly photoreceptor's structural restrictions, jointly govern light adaptation and provide reliable signalling at vastly varying light conditions.

We will first consider the challenge the natural scenes pose on light adaptation. We then highlight the importance for constructionist biomimetic approaches in modelling light adaption. Lastly, we review how adaptation innately arises from light information sampling. Because our focus is on the encoding of natural scenes as an animal locomotes, the adaptation mechanisms reviewed here operate fast (from milliseconds to about a second), and we exclude any subsequent longer term adaptations.

## Overview: the challenge to encode naturalistic inputs

**Figure 1:**
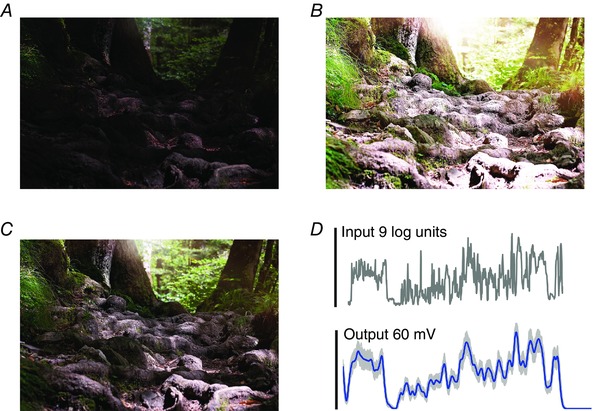
Biological photoreceptors achieve a larger dynamic range than a standard digital camera A standard digital camera cannot capture the whole range of light intensities in a natural scene with a single exposure. *A*, fine details of the ground are lost with a short exposure. *B*, longer exposures produce ‘white blobs’ in the picture due to saturation. *C*, the eyes can enhance detailed signals in the dark, and oppose saturation in bright light. *D*, a fly photoreceptor can encode vast light variations (9 log intensity units) into reliable neural responses within its limited output range (60 mV) without clipping them.

The dynamic range of a natural scene covers light intensities from the darkest shadows to the brightest reflections. Light intensity can vary many thousandfold in typical sun‐and‐shade scenes (Fig. 1 *A*), including landscapes against bright skies, window‐lit interiors with daylight outside and backlit objects (Rieke & Rudd, 2009). Therefore, a standard digital camera sensor, with output range of 256 values (8 bits), unavoidably fails to capture the full richness (Reinhard & Devlin, 2005). In a single exposure, either fine details in the shadows are lost through discretization, or bleached white blobs are produced by sensor saturation or clipping (Fig. 1 *B*).

Composite imaging techniques can be used to enlarge the dynamic range of cameras. For example, multiple exposures can be combined into a single picture, or, similarly, many graduated filters can be used for the picture integration (Nayar & Branzoi, 2003). However, these approaches work best either with static images or with specific landscape applications. And currently, there are no quick and efficient ways to extend the dynamic range for surveillance cameras to discriminate subtle light signal changes as the tracked objects move across different scenes (Dufaux, 2016).

Vision faces the same problem, with light intensity in natural habitats being an important cue for guided behaviours. With the eyes encoding their large natural input range so effortlessly, we are mostly oblivious of this challenge. Interestingly, however, the limited output range of diurnal photoreceptors is, in fact, not so different from that of a standard digital camera (Fig. 1 *C* and *D*) (Rieke & Rudd, 2009). If photoreceptors were linear encoders, their small signals to weak inputs would be corrupted by noise, while strong inputs would saturate. Here, output amplitude normalization was suggested as a general solution for encoding static image intensities (Laughlin, 1981). But to solve the problem of noise, which limits the reliable signalling range, photoreceptors and the following interneurons must, in fact, dynamically adjust their operational ranges to local light intensity changes (Laughlin, 1981; Zheng *et al*. 2009). Mechanistic understanding of how adaptation dynamics happen at the photoreceptor level would be important for making the next generation biomimetic light sensors, and a computational modelling approach can help in this task.

## Overview: the need for biomimetic models for phototransduction

Although photoreceptors’ great adaptability to different light stimulus statistics is well reported (Silva *et al*. 2001; Clark *et al*. 2013), detailed understanding of why and how this happens continuously has remained elusive. Theories based on various optimization criteria, including redundancy reduction (Attneave, 1954; Barlow, 1961), information maximization (Atick, 1992; van Hateren, 1992) and predictive coding (Srinivasan *et al*. 1982), have formulated this problem at an abstract neural output level. Generically, to maximize sensory information transfer, an optimal filter should change from a low‐pass integrator to a band‐pass differentiator with increasing stimulus signal‐to‐noise ratio (van Hateren, 1997). Whilst such filtering performance corresponds well with the adaptive trends in sensory‐neural signalling, the real neural outputs are more sophisticated, as they adapt continuously and near instantaneously to the temporal structure of stimuli. The theoretical filters, in contrast, are fixed, linear and optimized to Gaussian stimuli at given mean intensities (van Hateren, 1997). Thus, the computational link between the theories and the neural implementations of light adaptation has been incomplete at best.

This link is hard to capture by classic reductionist approaches, where models start from empirical mathematical descriptions (e.g. Volterra filter series and static nonlinearities), with parameters fitted to reproduce neural responses only for explicit stimulus conditions. The predictive power of such models is very limited, beyond the conditions in which the models were tested. To study the emergent properties of complex adaptive systems, such as living cells, it seems better to use bottom‐up biomimetic approaches, whereupon a computational virtual cell model is constructed to replicate its real counterpart's ultrastructure and signalling.

We recently constructed such a virtual *Drosophila* R1–R6 photoreceptor cell. Akin to a real R1–R6, this model integrates the parallel outputs of 30,000 G‐protein‐coupled receptor (GPCR) signalling pathways inside 30,000 microvilli (Hardie & Postma, 2008). The microvilli act as semi‐independent photon sampling and transduction units, which stochastically absorb incoming photons and adaptively transduce them to quantum bumps (QBs), summing up the macroscopic photoreceptor responses (stochastic adaptive sampling).

**Figure 2:**
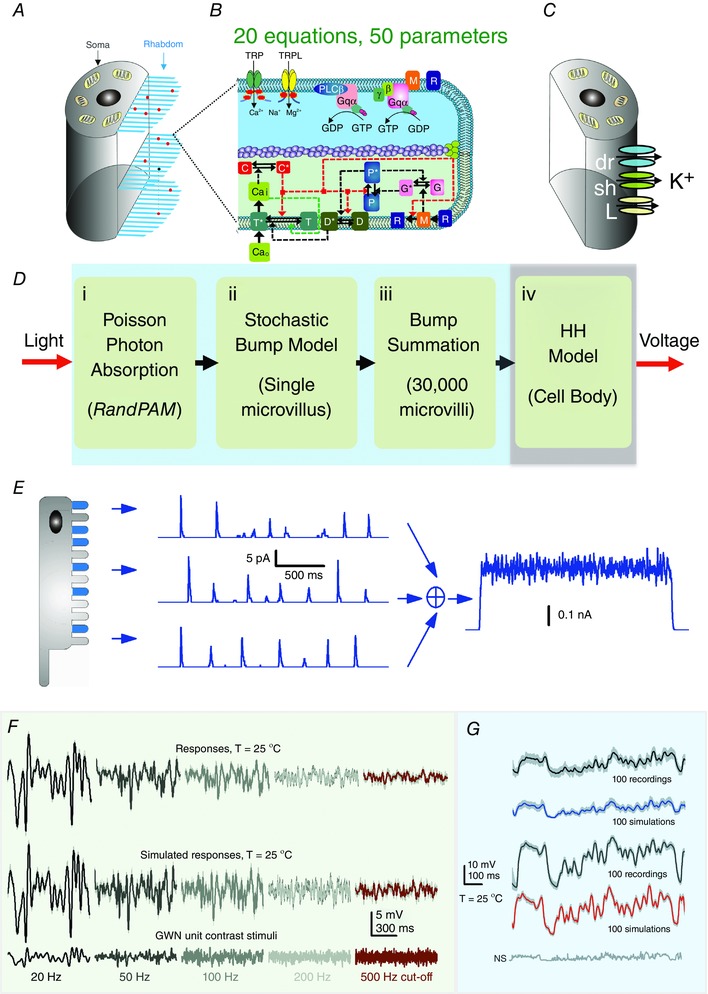
Schematic representation of the biophysical *Drosophila* photoreceptor model The complete model (Song *et al*. 2012) is composed of 4 biophysically realistic modules. The first three modules represent the phototransduction inside the rhabdomere, converting light input (a dynamic influx of photons) into the macroscopic output, light‐induced current (LIC). The fourth module models how the voltage‐sensitive conductances of the photo‐insensitive cell membrane shape the photoreceptor output. *A*, the rhabdomere contains 30,000 photon‐sampling units, microvilli (blue bristles). Random Photon Absorption Model (RandPAM) takes the total number of incoming photons and distributes them to the 30,000 microvilli, following Poisson statistics. *B*, each microvillus, which contains a G‐protein signalling cascade, can transduce single photon (red dots) energies into unitary responses, quantum bumps (QBs). The Stochastic Bump Model uses 20 equations with 50 parameters to simulate the phototransduction cascade. TRP, transient receptor potential ion channel; TRPL, transient receptor potential like. *C* and *D*, Hodgkin–Huxley Model transduces LIC into voltage response. This module models the dynamics of the voltage‐gated K^+^ conductances in the photo‐insensitive membrane (Niven *et al*. 2003). *E*, QBs from 30,000 microvilli sum the macroscopic LIC response. *F* and *G*, remarkably, the model generates realistic voltage output to any light intensity time series, including Gaussian white noise (GWN; *F*) and naturalistic stimulation (*G*) (Song *et al*. 2009, 2012, 2016; Song & Juusola, 2014; Juusola *et al*. 2015, 2016).

This process comprises four biophysically realistic submodules (Fig. 2 *D*) (Song *et al*. 2012; Juusola *et al*. 2015):

(i) Random Photon Absorption Model (RandPAM) distributes the incoming photons to the 30,000 microvilli following Poisson statistics (Fig. 2 *A*). Its output is the absorbed photon sequences of each microvillus (Song *et al*. 2016).
(ii) Stochastic Bump Model (Fig. 2 *B*): stochastic biochemical reactions inside a microvillus transduce the absorbed photon sequences to QB sequences (Pumir *et al*. 2008; Song *et al*. 2012).
(iii) Summation Model: QBs from 30,000 microvilli integrate to the macroscopic light‐induced current (LIC) response.
(iv) Hodgkin–Huxley Model of the photoreceptor plasma membrane (Fig. 2 *C*). This module transduces LIC into voltage response by reproducing the voltage‐gated K^+^ conductance dynamics on the photon‐insensitive membrane (Niven *et al*. 2003).

**Figure 3:**
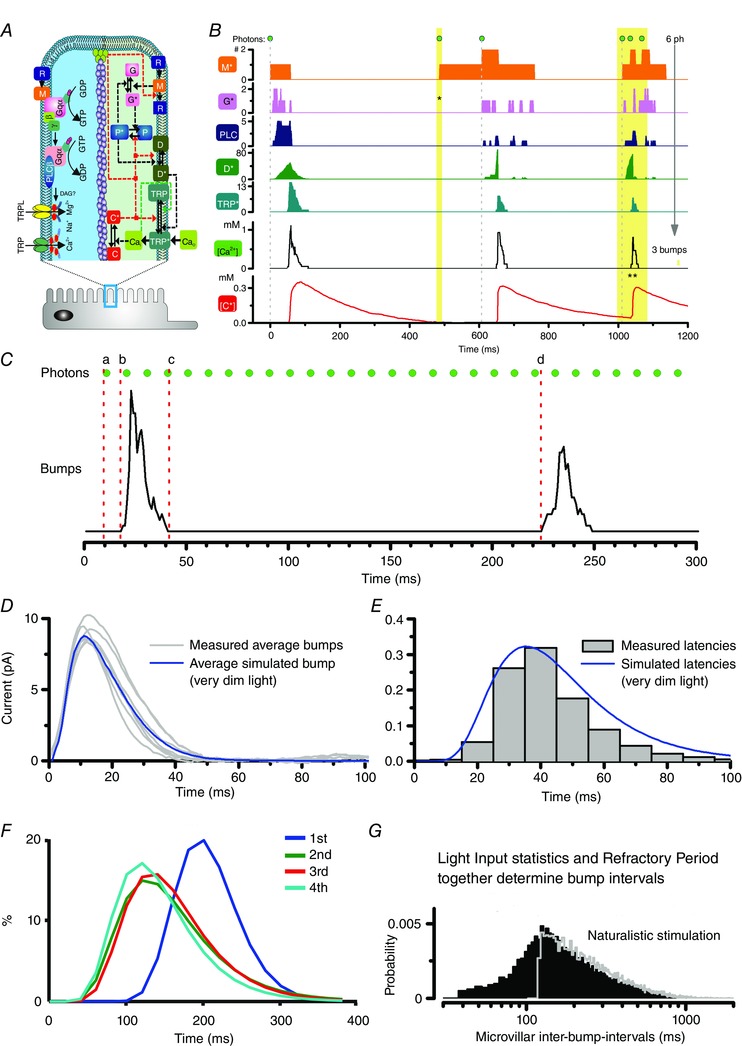
Quantal sampling factors in a single microvillus *A*, phototransduction cascade and the corresponding Stochastic Bump Model diagram. *B*, illustration of the molecular dynamics within the phototransduction cascade inside a single microvillus. Because of the local molecular feedbacks, not every absorbed photon evokes a QB (TRP^*^), e.g. the 2nd photon (1st yellow bar) does not result in a QB; the negative feedbacks, caused by the Ca^2+^ influx from the 1st QB, still inactivate the microvillus. The 3 photons in the 2nd yellow bar arrive so closely to the microvillus that they together only evoke one QB. *C*, key parameters of a QB sequence, including latency (delay between photon arrival to QB, *a* to *b*), QB waveform (*b* to *c*), and QB interval (*c* to *d*). *D*, measured and simulated average QBs in the dark. The inherently stochastic phototransduction cascade makes the QB shapes vary. *E*, measured and simulated latency distributions in dim conditions. *F*, simulated refractory period distribution for the 1st to the 4th bumps in a QB sequence. Refractory period distributions can only be estimated from the model simulations; they cannot be measured experimentally. *G*, simulated QB interval distribution when the photoreceptor responds to a naturalistic stimulus with a mean light intensity of 3 × 10^5^ photons s^−1^ (black). The grey line represents the QB interval distribution when the refractory period was used in the model as a fixed dead time (121 ms; Fig. 5 *C* in Song *et al*. 2012). The reason that the grey line still has a long tail in the distribution is because the microvillar QB intervals are determined by both the light input statistics and the refractory period distributions. The left half of the distribution is mostly determined by the refractory period distribution, while the grey long tail is defined by the stimulus statistics (Song & Juusola, unpublished results).

These modules were assembled step‐by‐step to simulate QB sequences of 30,000 microvilli and their dynamic integration (Figs 3 *A* and *B*). Parameters were not automatically fitted, but were fixed to their physiologically measured or pre‐estimated values. Remarkably, by comparing the response waveforms, signal‐to‐noise ratios and information transfer rates of the model simulations to the corresponding intracellular recordings, it has become clear that the model generates realistic voltage output to all tested light intensity time series (Fig. 2 *E* and *F*) without parameter refitting, even when the statistical structure of the stimulus changes (Song *et al*. 2009, 2012, 2016; Song & Juusola, 2014; Juusola *et al*. 2015, 2016). This would be impossible with the conventional reductionist modelling approach.

## Results: a stochastic adaptive sampling scheme from four quantal sampling factors

The model has helped us to elucidate how quantal information sampling underlies light adaptation in a fly photoreceptor (Song *et al*. 2012). In this scheme, four quantal factors (limitations) govern how light information is sampled (Fig. 3 *C*): (i) the number of sampling units (microvilli); (ii) sample size (QB waveform; Fig. 3 *D*); (iii) latency distribution (time delay between photon arrival to emergence of a QB; Figs 3 *E*) and (iv) refractory period distribution (time for a microvillus to recover after a QB; Fig. 3 *F*).

The basic rules about how these quantal factors curb light information sampling are as follows:

- A QB is the product of a successful photon transduction by a microvillus (Fig. 3 *A* and *B*). A QB is considered a sample of light, and its size and likelihood reflect the stimulus intensity.
- A single microvillus can produce only one QB at a time (Fig. 3 *B*) (Howard *et al*. 1987; Hochstrate & Hamdorf, 1990; Pumir *et al*. 2008; Song *et al*. 2012).
- After generating a QB, a microvillus becomes refractory (Fig. 3 *B*) (Scott *et al*. 1997; Liu *et al*. 2008). During the refractory period, the microvillus fails to produce a QB to a new photon hit (Hochstrate & Hamdorf, 1990; Song *et al*. 2012).
- Thirty thousand microvilli, which form the photoreceptor's light sensor (rhabdomere), sample incoming photons. QBs from all microvilli integrate the macroscopic response.
- QB sizes are reduced (amplitudes and durations) with brightening by both local and global feedback mechanisms. Light‐induced Ca^2+^ influx feeds back to multiple molecular targets in the microvillus, governing the QB termination and regulating the QB sizes (Nicol & Bownds, 1989; Yau, 1991; Reingruber *et al*. 2015; Hardie & Postma, 2008). Global feedbacks stem from global Ca^2+^ accumulation in the cell body and electromotive driving force attenuation through transient receptor potential (TRP)/transient receptor potential like (TRPL) channels in all microvilli. Brightening increases Ca^2+^ influx and photoreceptor depolarization, which strengthen the global feedbacks, shrinking QBs and compressing the macroscopic response more (Grzywacz & Hillman, 1988; Grzywacz *et al*. 1992).

We now assess how these rules jointly modulate a fly photoreceptor's output dynamics to light intensity time series.

## One QB at a time due to sublinear summation in phototransduction reactions

The light signal is quantal, with information carried by discrete photon arrivals. What is the quantal limit of vision? Or, how many photons must an eye capture for its beholder to see light? This question was raised already at the beginning of the 20th century (Bialek, 1987). Early psychophysical experiments indicated that humans perceive light when about seven quanta enter the eye (Hecht *et al*. 1942). Given that it is unlikely that these photons would hit the same photoreceptor, it was argued that a photoreceptor must detect single photons, which has recently been confirmed by experiments (Tinsley *et al*. 2016). Eventually, single photon responses, in the shape of analog current bumps, were measured both from single vertebrate (Baylor *et al*. 1979) and invertebrate photoreceptors (Yeandle, 1958; Henderson *et al*. 2000). As these responses were triggered by light quanta, they were named quantum bumps, representing the unitary end‐products of the phototransduction cascade.

Fly photoreceptors have the best‐studied phototransduction cascade, employing a prototypical G‐protein signalling pathway (Fig. 3 *A*) (Hardie & Postma, 2008). Upon a photon absorption, the activated rhodopsin (R^*^) activates the G protein, catalysing the exchange of GDP for GTP. This in turn produces the active Gα^*^‐GTP. Gα^*^‐GTP binds to PLC to form a G‐protein‐PLC complex, which hydrolyses phosphatidylinositol 4,5‐bisphosphate (PIP_2_) into diacylglycerol and inositol 1,4,5,‐trisphosphate (Hardie & Postma, 2008). Current evidence further suggests that the decrease of PIP_2_ concentration leads to membrane contraction and proton release, which together gate the Ca^2+^‐permeable TRP (and TRPL) light‐sensitive channels (Huang *et al*. 2010; Hardie & Franze, 2012; Hardie & Juusola, 2015). Powerful positive feedback, mediated by Ca^2+^ influx via the first activated TRP channels in the microvillus, facilitates rapid activation of its remaining channels. This floods the microvillus with up to ∼1 mm Ca^2+^ (Postma *et al*. 1999), which together with calmodulin (calcium buffer), forms various molecular feedbacks to terminate the QBs. The light‐induced opening of TRP/TRPL channels results in an influx of cations, e.g. Ca^2+^, Mg^2+^ and Na^+^, generating a ∼10 pA QB in dark‐adapted photoreceptors (Hardie & Postma, 2008). Essential cascade elements, including TRP/TRPL channels, are localized within a microvillus (Hardie & Postma, 2008).

The Stochastic Bump Model (the second module in our full model) can be used to simulate these molecular dynamics (Fig. 3 *B*) (Song *et al*. 2012). Previous biochemical phototransduction models simulate only single QBs (Pumir *et al*. 2008). In contrast, our model accommodates sequential photon absorptions, generating bump series. This is essential for studying continuous light adaptation processes.

**Figure 4:**
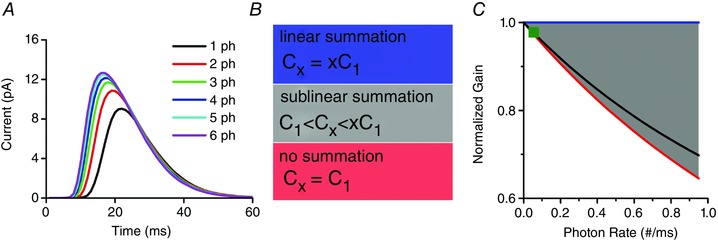
Sublinear bump summation *A*, only one QB is produced to simultaneous multi‐photon hits, with the resultant QB being smaller than the sum of those produced independently. *B*, the relationship between the bump charges for three different cases: linear summation, sublinear summation and no summation (*C_x_* is the *x*‐photon induced QB charge). *C*, the contribution of sublinear bump summation to light adaptation depends upon the photon arrival rate. Charges/photon is normalized to the single‐photon‐induced QB charge for the normalized gain. This normalized gain is from linear summation, but it varies nonlinearly with the photon rate for sublinear summation (grey area) and no summation (red line). The black line is an example produced by our Stochastic Bump Model. Sublinear bump summation contributes to light adaptation marginally in a fly photoreceptor (<1%, green square).

From the model simulations, we know that phototransduction is highly nonlinear. Sublinear bump summation can happen when more than one photon hits the same microvillus at once (or within the time resolution of a QB). Multiple rhodopsins can be activated, but only one QB is produced (3rd photon in Fig. 3 *B*), with the resultant QB being smaller than the sum of those produced independently (Fig. 4 *A*) (Pumir *et al*. 2008).

Sublinear bump summation could reduce the QB/photon gain by severalfold, and so contribute to light adaptation (Fig. 4 *B* and *C*) (Pumir *et al*. 2008). We recently deduced that such a contribution would depend upon the likelihood of simultaneous multi‐photon hits (Song *et al*. 2016), as determined by RandPAM (the first module of our full model). Our calculations revealed that sublinear bump summation in a fly photoreceptor contributes to light adaptation only marginally (Fig. 4 *C*), since a typical fly photoreceptor has tens of thousands of microvilli (Boschek, 1971), each of which rarely experiences simultaneous multi‐photon hits, even in bright daylight (≤ 1%, green square) (Song *et al*. 2016). However, for a photoreceptor with significantly fewer sampling units, such as the stick insect (*Carausius morosus*) (Frolov *et al*. 2012), multi‐photon hit‐induced gain control may affect light adaptation more.

## Refractory period: beyond photon counting

Past experimental results have suggested that, after generating a QB, phototransduction reactions remain briefly in an inactive state, analogous to refractoriness in action potential dynamics (Baehr & Palczewski, 2002). Even though a refractory period could not be directly measured, experiments suggested that it only lasts for a short moment, as a second intense bright flash did not excite a response if given within 50–100 ms of the first one (Hochstrate & Hamdorf, 1990). But since this effect was only observed in very bright light conditions, when all microvilli were potentially activated, the results suggested that reactions inside individual microvilli had become refractory (Hochstrate & Hamdorf, 1990). Additional experiments on fly mutants with reduced calmodulin concentration were supportive of refractoriness affecting QB production (Scott *et al*. 1997; Liu *et al*. 2008).

Our stochastic bump model simulations have now clarified how phototransduction reactions inside the light‐activated microvillus remain refractory after generating a QB (Song *et al*. 2012). Refractoriness, in fact, is an emergent (intrinsic) property of the Stochastic Bump Model in response to a photon sequence. As soon as a QB is generated, the negative Ca^2+^ and calmodulin feedbacks hold the microvillus in a state of inhibition, during which it cannot respond to subsequent photons. The length of this refractory period is set by the dynamic balance between the positive and negative molecular feedbacks. Only after the negative feedbacks have relaxed enough can new photon arrivals trigger responses with positive feedbacks outgrowing the effects of inhibition (Song *et al*. 2012).

**Figure 5:**
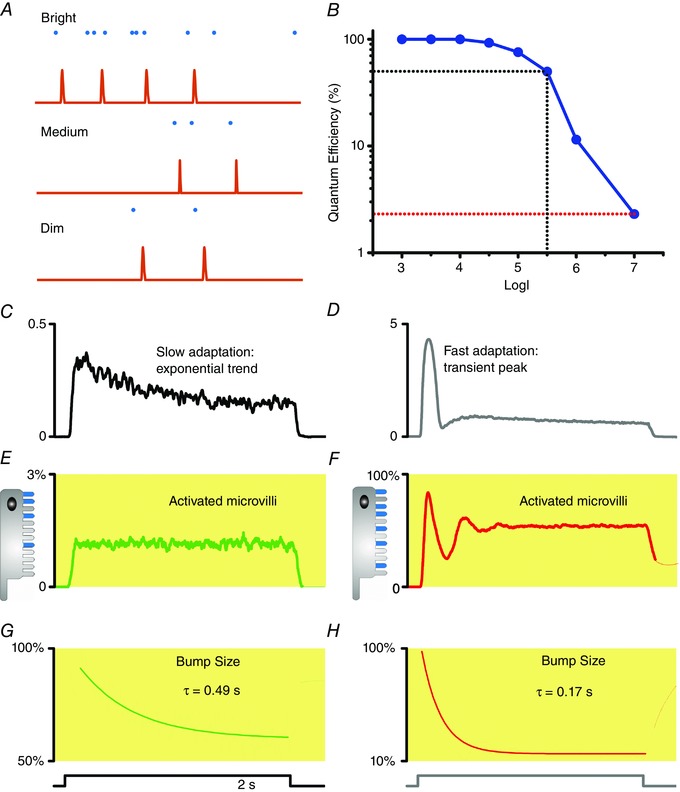
The roles of refractory period to light adaptation *A*, the event rate (illustrated by unitary QBs) in a microvillus depends on the incoming photon rate. In dim conditions, photon arrivals to an individual microvillus are rare and virtually every photon hit (absorption) evokes a QB. With increasing photon rate (medium light), the quantum efficiency (photon to bump conversion probability) decreases gradually. In very bright daylight, photon arrivals can be so frequent that the refractory period effectively sets the maximum QB production rate (sample rate). *B*, quantum efficiency changes automatically with brightening from 100% to 1%, acting as a powerful gain control mechanism. *C*, a fly photoreceptor's LIC response to a dim light pulse. *D*, a fly photoreceptor's LIC response to a bright light pulse. *E*, during a dim light stimulus, the QB count (samples) from the activated microvilli does not show a fast adapting peak. *F*, in response to a bright pulse, the QB count (samples) from the activated microvilli first peaks, then rapidly falls, before settling to a steady‐state as the photon arrivals and refractory periods balance. *G* and *H*, QB size must reduce over time to account for the slow exponential response trend. The receptor current displayed in *C* and *D* can be simulated by taking account of both factors: the reduction of activated microvilli number (*E* and *F*), and QB size reduction (*G* and *H*) (Song *et al*. 2012).

Furthermore, model simulations have elucidated how refractoriness contributes to light adaptation (Song *et al*. 2012; Song & Juusola, 2014). Refractory microvilli provide a powerful automatic gain control mechanism (Teich & Lachs, 1983; Song *et al*. 2012; Juusola *et al*. 2015). The refractory period (Fig. 3 *G*), together with the photon arrival rate (Fig. 5 *A*), jointly determines the microvillar QB production rate. In dim conditions (Fig. 5 *A*, Dim), photon arrivals are sparse. Therefore, photon hits to an individual microvillus are very rare (Song *et al*. 2016), generating a QB from virtually every absorbed photon (Song *et al*. 2012). With an increasing photon rate, the quantum efficiency (photon to bump conversion probability) decreases gradually (Fig. 5 *A*, Medium) (Song *et al*. 2012). In very bright daylight (Fig. 5 *A*, Bright), photon arrivals are so frequent that the refractory period effectively sets the maximum QB rate (sample rate) (Hochstrate & Hamdorf, 1990; Song *et al*. 2012). Thus, as more and more microvilli become refractory with brightening, quantum efficiency changes automatically from 100% to 1%, providing a powerful gain control mechanism (Fig. 5 *B*).

Importantly, refractoriness also dynamically attenuates photoreceptor output to salient light inputs (Teich & Lachs, 1983; Song *et al*. 2012; Song & Juusola, 2014; Juusola *et al*. 2015). A bright light onset evokes a large transient response. This results from fast adaptation (Fig. 5 *D*). The response first rapidly decays and then plateaus (Fig. 5 *C* and *D*), even when the stimulus stays the same (light step). The fast adaptation reflects refractory QB production by the limited microvillus pool (30,000 in *Drosophila*). At a bright stimulus onset, most microvilli of a dark‐adapted photoreceptor are available, producing their first QBs with high quantum efficiency. But this makes them also refractory, leaving a smaller pool of microvilli available for responding to the next photons. Thus, the QB count (samples) from the activated microvilli first peaks, then rapidly falls, before settling to a steady‐state as photon arrivals and refractory periods balance (Fig. 5 *F*). However, the early transient response cannot be evoked by dim continuous stimulation as most microvilli are available (few are refractory) to respond to fewer photon arrivals (Fig. 5 *E*). These dynamics are further shaped by the concurrent QB size adaptation; the brighter the stimulation the smaller the QBs (see below).

Thus, refractory sampling dynamically accentuates QB rate changes in photoreceptor output. This can enhance the neural representation of phasic information against any static background, such as line elements and contrast edges of natural scenes (Juusola & de Polavieja, 2003; Song & Juusola, 2014). Regulation of sample numbers (quantal responses) by refractoriness is likely to be a general adaptation mechanism that affects also other sensory neurones, including mechanoreceptors (Song *et al*. 2015).

## The size of the microvillus (sampling unit) population limits encoding

Rhabdomeres of R1–R6 photoreceptors of different fly species boast different microvilli numbers; a typical R1–R6 of a slow‐flying *Drosophila* has 30,000 microvilli whilst that of a fast‐flying *Calliphora* has 90,000. So how does the size of a photoreceptor's microvillus population affect its light information capture?

In our model, we consider each microvillus a photon sampling unit, as suggested by experimental results (Hochstrate & Hamdorf, 1990; Juusola & Hardie, 2001a, b; Song *et al*. 2012, 2016). We further assume that the microvilli transduce their photon absorptions to QBs independently. Although a single microvillus can only produce a discrete QB sequence (Song *et al*. 2012), the QB sequences from all the microvilli sum up the graded macroscopic LIC. This simple QB summation generates realistic LIC responses, in which information transfer closely approximates that of the corresponding intracellular recordings to given test stimuli.

Therefore, the number of microvilli (photon sampling unit) is a key parameter that limits a photoreceptor's encoding capacity (Howard *et al*. 1987; Hochstrate & Hamdorf, 1990; Song *et al*. 2012). Theoretically, the photoreceptors with the most and fastest microvilli should produce the output with the highest fidelity (Juusola & Hardie, 2001a,b; Gonzalez‐Bellido *et al*. 2011), and this is indeed what the simulations show (Song & Juusola, 2014). If a photoreceptor had an infinite number of sampling units, each with the briefest refractory period, its macroscopic LIC would be a linear summation of QBs. However, photoreceptors transform light intensity changes to QB rate changes in a highly nonlinear manner, which is dynamically determined by the spread of refractoriness within their limited microvilli population. The fewer the microvilli and the longer their refractoriness, the more photons a photoreceptor would lose in bright stimulation and the lower the intensity, which would saturate its macroscopic response. Thus, the photoreceptor structure (microvillus population size) reflects an evolutionary trade‐off between the animal's visual needs and the cost of sampling (Song & Juusola, 2014).

In summary, the limited microvillus population and its refractoriness makes a photoreceptor an imperfect photon counter (Burns & Arshavsky, 2005; Juusola *et al*. 2015). Only in dim light are photon arrival intervals in a microvillus much longer than its refractory period and the photoreceptor's photon absorption rate can be estimated by counting its QBs. Interestingly, however, losing most photons to refractory microvilli in extreme daylight (10^6^–10^9^ photons s^−1^) is not critical for good vision. For example, as long as a *Drosophila* R1–R6 photoreceptor ‘counts’ 5 × 10^4^–5 × 10^5^ quantum bumps s^−1^, its macroscopic response would have a very high signal‐to‐noise ratio (Song & Juusola, 2014).

## Benefits of stochastic sampling: QB shuffling through stochastic QB timings

Phototransduction reactions are inherently stochastic, due to the low number of molecules involved. In the past, the QB variations have been considered mostly noise that lowers a photoreceptor's information transfer (Lillywhite, 1979; Lillywhite & Laughlin, 1979; Laughlin & Lillywhite, 1982). However, the model simulations suggest that light adaptation benefits from stochasticity in quantal information sampling. Similar to what is seen in real photoreceptor outputs (Faivre & Juusola, 2008; Zheng *et al*. 2009), simulations have shown that variable QBs from a large microvillus population sum up largely invariable response waveforms to naturalistic stimuli at different illumination conditions (Song *et al*. 2012). This property directly emerges from the stochastically modelled phototransduction reactions. Rather than treating stochasticity simply as additive noise, as is done in conventional modelling approaches, it is critical to capture the stochastic phototransduction dynamics. We employed a Gillespie algorithm to explicitly simulate each molecular reaction inside each of the 30,000–90,000 microvilli (Gillespie, 1976), causing realistic variations in their QB waveforms and timings.

**Figure 6:**
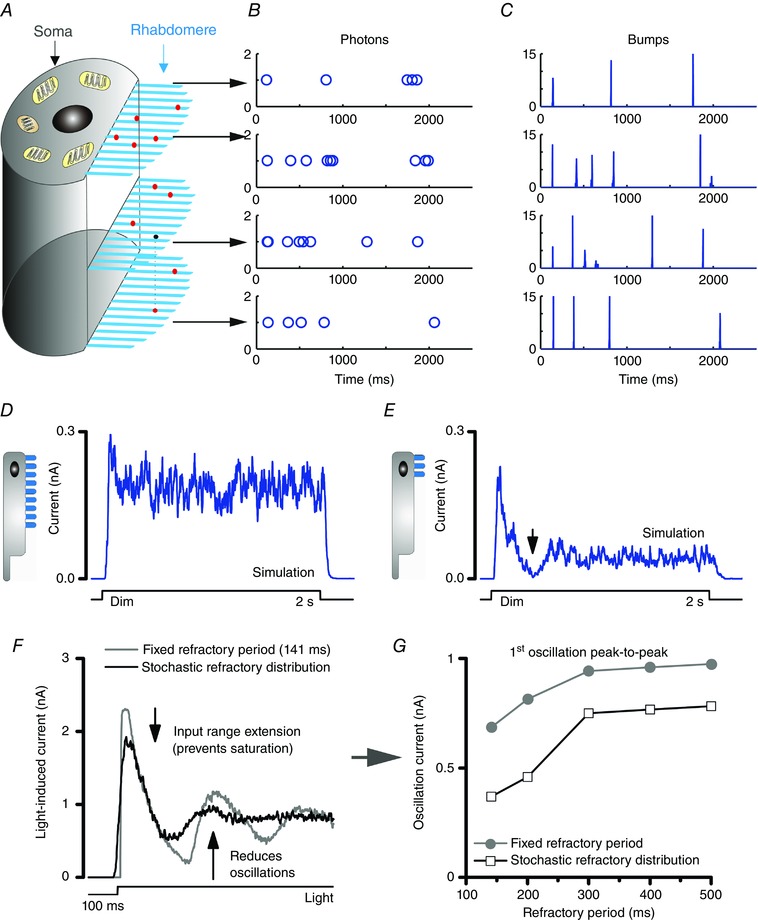
The roles of stochasticity in photoreceptor encoding *A*, 30,000 microvilli in a photo‐sensitive rhabdomere sample incoming photons. *B*, stochastic photon arrivals to a microvillus population. *C*, variable QB waveforms and timings in a microvillus. *D* and *E*, stochastically operating microvilli resist saturation. Responses to a dim light pulse are not saturated even if a photoreceptor had only very few microvilli, e.g. 3,000 microvilli (*D*), or 300 microvilli (*E*). Even when the microvillus number is reduced by 100 times to 300, it is hard to knock out all microvilli at once, because there are always some returning to the pool of available ones at any one moment (responses at black arrow is not flat zero). *F* and *G*, stochastic latencies and refractory periods help to prevent saturation and reduce oscillations in photoreceptor output, in comparison to that resulting from a fixed refractory period (black arrows).

We coined the term *stochastic sampling* to describe stochastic photon absorptions and the QB conversions of the entire microvillus population. There are two important aspects to this: (i) the stochastic photon arrivals to the microvillus population (Fig. 6 *B*) (Song *et al*. 2016), and (ii) the variable QB waveforms and timings (Fig. 6 *C*) (Stieve & Bruns, 1983; Kirkwood & Lisman, 1994; Henderson et al. 2000; Juusola & Hardie, 2001a; Pumir *et al*. 2008; Song *et al*. 2012). Both of these aspects affect photoreceptor output dynamically. The stochastically operating microvilli resist saturation in generating the macroscopic photoreceptor output (Song *et al*. 2012; Juusola *et al*. 2015). It is hard to knock out all microvilli at once, when there are always some returning to the pool of available ones in any one moment (Fig. 6 *D* and *E*). Sampling with equal probabilities utilizes microvilli and photoreceptor output range more evenly (Song *et al*. 2016). Furthermore, QBs are effectively shuffled in time by stochastic latencies (Stieve & Bruns, 1983; Juusola & Hardie, 2001a,b; Faivre & Juusola, 2008; Song *et al*. 2012). This contributes to weighting microvilli output and to evoking responses with similar temporal resolutions in different illumination conditions. Stochastic sampling may in fact represent a generic solution to the temporal aliasing problem (Song *et al*. 2012; Juusola *et al*. 2015). Simulations show that stochastic refractory periods reduce oscillations in photoreceptor output in contrast to those seen in models with a fixed refractory period (Fig. 6 *F* and *G*) (Stieve & Bruns, 1983; Song *et al*. 2012). A more detailed account of how stochastic sampling benefits encoding and the related trade‐off between antialiasing and broadband noise can be found in Juusola *et al*. (2015) and Juusola & Song (2017).

## Bump adaptation due to local and global calcium feedbacks

Noise analysis has indicated that QB waveforms adapt to ongoing light conditions, becoming smaller and briefer with brightening (Dodge *et al*. 1968; Wu & Pak, 1978; Wong & Knight, 1980; Wong *et al*. 1982; Juusola *et al*. 1994; de Ruyter van Steveninck & Laughlin, 1996; Juusola & Hardie, 2001a; Burton, 2006). This adaptive bump size reduction was originally deduced by reverse inference with the central assumption being that the macroscopic response is a linear summation of QBs. Experiments have further shown that QBs get smaller when intracellular or extracellular [Ca^2+^] is elevated, reflecting changes in light intensity. Our stochastic sampling models add further feed‐forward evidence for QB adaptation (Song *et al*. 2012).

**Figure 7:**
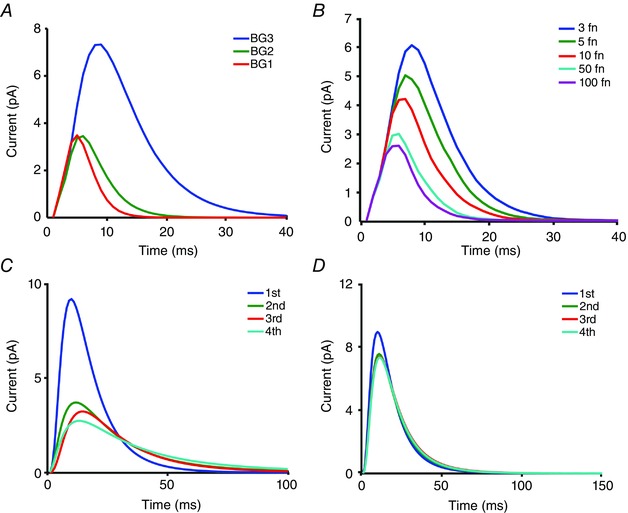
Bump adaptation in the model *A*, 1st QBs in response to naturalistic stimulation at different mean light intensities. Light intensities are 3 × 10^5^, 3 × 10^4^ and 3 × 10^3^ photons s^−1^ per photoreceptor for BG1, BG2 and BG3, respectively. QBs become smaller and briefer with brightening. *B*, the QB size reduction is achieved by increasing a feedback parameter, *fn*, which tunes the inhibition strength of Ca^2+^. *C* and *D*, 1st to 4th QBs in a single microvillus shows that QBs adapt over time, with the 1st QBs being the largest. The amount of QB size reduction, however, depends upon the photon arrival rate. QB sizes over time are reduced more with more frequent photon arrivals (5 photons s^–1^ per microvillus in *C*), but less with sparse photon arrivals (2 photons s^–1^ per mirovillus in *D*).

To replicate the recordings, the mean QB size in simulations must change at different light conditions (Fig. 7 *B*), reducing up to 50 times from dim to very bright illumination (Fig. 7 *A*) (Juusola & Hardie, 2001a,b; Song *et al*. 2012). The QB size reduction is achieved by increasing a feedback parameter, which tunes the inhibition strength of Ca^2+^ (Fig. 7 *A*). Both the local Ca^2+^ influx inside a microvillus and the global somatic Ca^2+^ spread from many microvilli can increase inhibition (Hardie & Postma, 2008; Song *et al*. 2012).

Model simulations further suggest that QB size must adapt over time (Fig. 7 *C* and *D*) (Song *et al*. 2012). When stimulated with a bright step‐stimulus, a real LIC peaks and then rapidly decays toward a lower plateau. But, if all QBs were identical, the macroscopic LIC would simply represent the number of activated microvilli at a given photon rate, with a flattened steady‐state. Thus, the model must progressively strengthen the Ca^2+^‐feedbacks to reduce QB waveforms over time (Fig. 5 *G* and *H*). This memory effect induces a temporal adaptation that improves the signal‐to‐noise ratio of macroscopic responses, in comparison to the estimates that were sampled randomly (Song *et al*. 2012).

## Contrast normalization due to global voltage feedbacks

A fly photoreceptor is functionally divided into two parts, a photo‐sensitive rhabdomere and a photo‐insensitive cell body. The QB dynamics, as reviewed above, happen in the rhabdomere. But what is the role of the cell body in information processing? The cell body membrane contains a suite of voltage‐gated K^+^ channels, in which response dynamics can be modelled using classic Hodgkin–Huxley formalism (Hodgkin & Huxley, 1952; Weckström & Laughlin, 1995; Niven *et al*. 2003; Vähäsöyrinki *et al*. 2006). In the past, adaptive shunting by voltage‐sensitive K^+^ conductances was considered a major cause for light adaptation (Weckström *et al*. 1991). While our quantal information sampling scheme has now established that the major adapting factors reside in photon sampling, voltage‐sensitive K^+^ conductances, nevertheless, interact in a nonlinear way with the rhabdomeric light‐gated conductances in shaping the photoreceptor voltage output.

In our models, the voltage responses are generated by injecting the macroscopic LIC to a Hodgkin–Huxley model of the photoreceptor cell body membrane. These voltages simultaneously regulate the electromotive driving force of LIC through TRP/TRPL channels, as a global feedback. Although the concept of regulating an ion channel's driving force by voltage is not new (Hodgkin & Huxley, 1952), how this influences adaptation, especially to naturalistic stimulation, was less clear. Simulations showed that the voltage‐sensitive membrane acts as a dynamic gain controller with some interesting properties.

**Figure 8:**
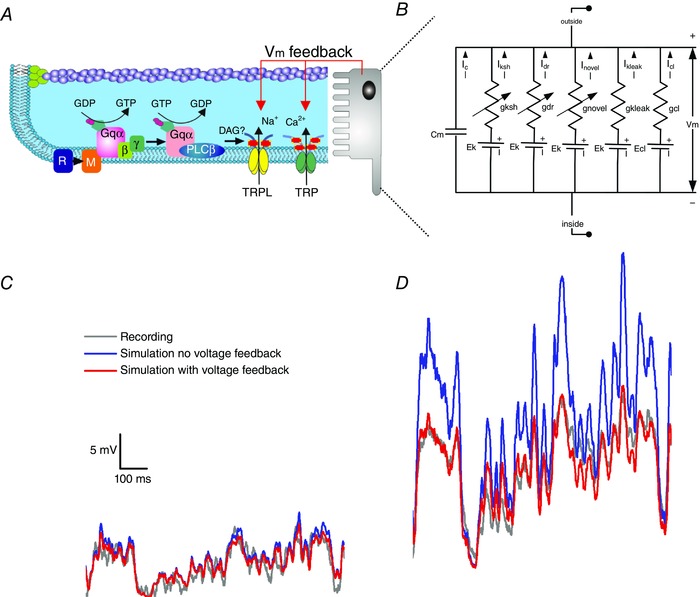
Contrast normalization by global voltage feedbacks *A*, QB dynamics take place in the microvilli of the rhabdomere. *B*, Hodgkin–Huxley formalism is used to model a suite of voltage‐gated K^+^ channels on the R1‐R6 photoreceptor body. The simulated voltage responses were generated by injecting macroscopic LICs to the Hodgkin–Huxley model of the photoreceptor membrane. The macroscopic voltage response acts as a global feedback, regulating the electromotive driving force through all the TRP/TRPL channels. *C* and *D*, the global voltage feedback is adaptive, compressing the signal less with dim stimulation (*C*), but more with bright stimulation (*D*).

First, it provides a global negative feedback. The total LIC of the microvillus population charge up the photoreceptor voltage, which in return reduces the electromotive force for every single TRP channel in every microvillus (Fig. 8 *A* and *B*) (Song *et al*. 2012). Second, the regulation is adaptive (Fig. 8 *C* and *D*). The brighter the light input, the higher the membrane voltage, the lower the electromotive force and the smaller the generated QBs. Third, it contributes to the relative contrast normalization of the responses to naturalistic light contrast time series stimuli at different illumination conditions (Song *et al*. 2012) (Fig. 8 *C* and *D*). The voltage feedback compresses signals less in dim conditions, but far more in bright stimulation, comparable to divisive nonlinearity (Heeger, 1992). The difference here is that the normalization is achieved through a global feedback within a single photoreceptor, rather than by a divisive operation of nonlinear input summation from many neurons. Finally, this global feedback has little influence on the temporal resolution of photoreceptor output. Experiments have shown that the photoreceptor membrane impedance has a broader bandwidth than the corresponding LIC (Juusola & Weckström, 1993; Juusola & Hardie, 2001a,b). Thus, as the voltage feedback neither clips the LIC frequencies significantly nor seems to produce much additional noise, it leaves the output information practically intact (Juusola & de Polavieja, 2003; Song *et al*. 2012; Song & Juusola, 2014; Juusola *et al*. 2015).

## Other mechanisms

Besides the four quantal information sampling factors, fly photoreceptors reveal further mechanisms that can contribute to light adaptation (Lan *et al*. 2012). These include adaptive shunting by voltage‐gated conductances (Niven *et al*. 2003), lateral and temporal inhibition by synaptic feedbacks (Srinivasan *et al*. 1982; Zheng *et al*. 2009; Kramer & Davenport, 2015), intracellular pupil mechanisms (Franceschini & Kirschfeld, 1976), metabolic energy constraints (Niven *et al*. 2007), and photomechanical photoreceptor contractions (Hardie & Franze, 2012; Juusola *et al*. 2016).

## Conclusions and remarks

In summary, we have explained how light adaptation in fly photoreceptors emerges through a stochastic adaptive sampling framework. In this framework, light adaptation is largely accountable by two quantal sampling mechanisms: (i) reduction in sample numbers (QBs from activated microvilli) and (ii) sample sizes (QB waveforms), each contributing about 50% at normal daylight levels (10^5^ photons s^−1^). Refractory sampling automatically tunes quantum efficiency (photon to QB conversion probability) at different light levels. In dim conditions, quantum efficiency is near 100%, providing highly sensitive vision. Yet, quantum efficiency drops gradually with brightening, reaching ≤ 1% in bright daylight. Conversely, QB size reduction, through Ca^2+^ and voltage feedbacks, improves temporal resolution and increases contrast gain in photoreceptor output. Stochastic QB integration (from the entire microvillus population) makes the resulting voltage responses to the same naturalistic contrast stimulus look similar in different light conditions.

The four quantal sampling factors, microvillus numbers, refractory period, QB latency and size variations, have been known for some time. But only recently, by integrating a stochastic adaptive sampling model, could we methodically work out how light adaptation in fly photoreceptors emerges from these limits, proving a large dynamic range. Equally importantly, this framework predicted how the same quantal factors govern the photoreceptors’ signalling performances in different slow‐ and fast‐flying fly species, matching vision to different lifestyles through evolution (Song *et al*. 2012). In these models, the emergent properties of stochastic adaptive sampling stem naturally from thousands of realistically operating G‐protein signalling cascades.

We believe that stochastic quantal adaptive sampling provides a general evolutionary strategy for reliable sensory information encoding. The stochastically operating sampling units could be anything from individual cells or synapses even to ion channels. For example, having a large population of refractory ion channels can induce the adaptive dynamics of a mechanoreceptor (Song *et al*. 2015). In the future, similar sampling frameworks may help us to understand how synaptic and neuronal clusters process information.

## Additional information

### Competing interests

The authors declare no competing interests.

### Author contributions

Z.S. and M.J. wrote the paper. M.J. and Z.S. approved the final version of the manuscript and agree to be accountable for all aspects of the work. All persons designated as authors qualify for authorship, and all those who qualify for authorship are listed.

### Funding

Z.S. thanks EPSRC‐funded 2020 Science fellowship (EP/I017909/1) for funding. M.J. thanks these funding sources for supporting this work: the State Key Laboratory of Cognitive Neuroscience and Learning open research grant, Natural Science Foundation of China Project 30810103906, Jane and Aatos Erkko Foundation Fellowship, Leverhulme Trust Grant RPG‐2012‐567, and Biotechnology and Biological Sciences Research Council Grants BB/F012071/1, BB/D001900/1, BB/H013849/1 and BB/M009564/1.
